# NMDA receptors and L-arginine/nitric oxide/cyclic guanosine monophosphate pathway contribute to the antidepressant-like effect of Yueju pill in mice

**DOI:** 10.1042/BSR20190524

**Published:** 2019-09-16

**Authors:** Wei Wang, Tong Zhou, Rong Jia, Hailou Zhang, Yi Zhang, Chunxiu Wang, Yuwei Dong, Jianghui Wang, Li Sheng, Haoxin Wu, Gang Chen, Wenda Xue

**Affiliations:** 1 Key Laboratory of Integrative Medicine for Brain Diseases, School of Basic Biomedical Science, Nanjing University of Chinese Medicine, Nanjing 210023, China; 2 School of First Clinical Medicine, Nanjing University of Chinese Medicine, Nanjing 210023, China; 3 School of Psychology, Nanjing University of Chinese Medicine, Nanjing 210023, China; 4 School of Medicine and Life Sciences, Nanjing University of Chinese Medicine, Nanjing 210023, China; 5 Co-innovation Center of Neuroregeneration, Nantong University, Nantong 226001, China; 6 Interdisciplinary Institute for Personalized Medicine in Brain Disorders, and Research Center for Formula and Syndromes, Jinan University, Guangzhou 510632, China

**Keywords:** antidepressant-like effect, cGMP, NMDA, NO, Yueju pill

## Abstract

The present study aims to evaluate the involvement of N-methyl-d-aspartate receptor and nitric oxide (NO)/cyclic guanosine monophosphate (cGMP) system in antidepressant-like effects of Yueju pill (YJ), a Chinese herbal medicine. The immobility time in tail suspension test (TST) and forced swim test (FST) was used to assess the antidepressant effects. Prior administration of L-arginine (750 mg/kg, intraperitoneal [i.p.]), a NO synthase substrate that enhances NO signaling or sildenafil (5 mg/kg, i.p.), a phosphodiesterase 5 inhibitor that enhances cGMP, blunted the antidepressant-like activity of YJ (2.7 g/kg, i.g.). Co-treatment of ineffective dose of YJ (1.35 g/kg, i.g.) with one of the reagents that suppress the NO/cGMP signaling, including methylene blue (10 mg/kg, i.p.), an inhibitor of NO synthase; 7-NI (7-nitroinidazole, 30 mg/kg, i.p.), an nNOS specific inhibitor; L-NAME (10 mg/kg, i.p.), a non-specific inhibitor of NO synthase; and MK-801 (0.05 mg/kg, i.p.), an NMDA receptor antagonist, reduced the immobility time in TST and FST, compared with those in vehicle or single drug treatment groups. Neither above drugs alone or co-administrated with YJ affected locomotor activity or anxiety behavior in open field test. Thus, our results suggest that the antidepressant-like action of YJ may depend on the inhibition of NMDA/NO/cGMP pathway.

## Introduction

Major depressive disorder (MDD) is a serious, disabling, and chronic recurring psychiatric illness that affects millions of individuals worldwide [1], and has a significant negative impact on public health [2]. Currently, the present finding indicated that in the brain as well as in the plasma of MDD patients, the glutamate levels were increased compared with those in normal controls. [3–5]. Meanwhile, blockade of NMDA receptors displayed antidepressant activity, suggesting that glutamate played an important role in the depressive-like behaviors [6–12]. A typical non-competitive NMDA receptor, ketamine was demonstrated rapid antidepressant effect in both patients and rodents, especially in MDD or bipolar disorder [11,13]. Additionally, the conventional antidepressants: SSRIs (selective serotonin reuptake inhibitors) had intense effect on NMDA receptor activity and could affect depressive behavior in MDD patients [14,15]. Recently, we found that the ethanol extract of Yueju pill (YJ), a traditional Chinese medicine, showed rapid antidepressant-like effect in acute and chronic animal models [16], and chronic YJ could improve the depressive behavior in patients [17]. Previous studies showed YJ as well as ketamine, both reduced expression of NR1 subunit of NMDA receptor [18], which was associated with the antidepressant activity. It remains to determine the role of NMDA signaling in antidepressant effect of YJ.

Recent researches supported that the NMDA-NO-cGMP signaling pathway involved in antidepressant effects. Nitric oxide synthase (NOS) catalyzed the synthesis pathway from L-arginine to NO and its activity was induced by NMDA receptor [19,20]. SSRIs also developed antidepressant activity via blockage of NOS activity in rodents [21–24]. NO had been characterized to target the soluble guanylate cyclase (sGC), while the sGC could convert guanosine 5-triphosphate (GTP) to cyclic guanosine 3,5-monophosphate (cGMP) [25]. Meanwhile, NO was also important in the CNS system and it participated in neurotransmitter release, thus affecting synaptic plasticity [21]. Recently, some chemical compound displayed antidepressant-like effect associated with decreased NO level; it indicated that NO may play a key role in depression [26,27]. NO was also regarded as a modulator for the concentration of cGMP, which was implicated in the phosphorylation of CREB, while it was an important target gene of antidepressants [21,28]. Therefore, NO/cGMP pathway is suggested to be involved with the pathophysiology of depression and regulation of such signaling may represent a new target to a pharmacological approach [20,26,29,30].

In the present study, we tested the role of NMDA/NO/cGMP signaling in the antidepressant-like effect of YJ. Here, we hypothesized that down-regulation of NMDA/NO/cGMP pathway could induce the antidepressant-like activity of YJ. Based on our results, we suggest that YJ’s antidepressant response is mostly depend on NMDA/NO/cGMP signaling pathway.

## Materials and methods

### Animals

Adult male Balb/cJ mice, aged 12–13 weeks, were obtained from Shanghai Sippr-BK laboratory animal Co. Ltd. in China, within the range of 23–25 g. All animals were kept under standard conditions of temperature 23 ± 2°C and light/dark cycle of 12 h each. Free access to food and water was given except for the period of experimentation outside the cage. Four to six animals were housed in a single cage. Full efforts were paid to conduct the experiments within 09:00 am to 15:00 pm. In each group, there were eight to ten animals and experiments were performed by strictly following the Institutional Guideline of Animal Care and Use Committee (Key Laboratory of Integrative Medicine for Brain Diseases, Nanjing University of Chinese Medicine). In each independent behavior test, about 32–40 animals were used. All animal procedures were carried out in accordance with the Guide for the Care and Use of Laboratory Animals approved by the Institutional Animal Care and Use Committee at Nanjing University of Chinese medicine. For all behavioral testing, mice were weight matched and used only once. The experimenters were blind to the assignments of the mice to the experimental groups. The treatment and behavior test of mice were in the key Laboratory of Integrative Medicine for Brain Diseases, Nanjing University of Chinese Medicine.

### Tail suspension test

Mice were assessed in TST, which was performed with a computerized device that allowed four animals to be tested at one time. In a chamber both acoustically and visually isolated, an individual mouse was suspended 50 cm above the floor by adhesive tape placed approximately 1 cm from the tip of the tail. The activities of animals were videotaped. ANY-maze software (Stoeling Co.Ltd., U.S.A.) was used to calculate the total time spent immobile during the last 4 min in a 6-min testing period [16,31,32].

### Forced swimming test

FST is also a widely used paradigm for behavioral despair and antidepressant response in rodents. Mice were removed from their home cages, placed individually into a clear glass tank (40 cm high and 20 cm in diameter) which filled with 30 cm of water (22–23°C), and allowed to swim for 6 min [16,33]. At the end of the test, the animals were removed from the water, dried with a paper towel, and returned to their home cages. The mice were considered immobile when floating in the water without struggling and making only those movements necessary to keep their heads above the water. Total immobility times during the last 4 min of the 6-min testing period were recorded by ANY-maze software.

### Open filed test

OFT assesses locomotor activity and anxiety-like behavior in a bright-lit open area. Testing was performed for 5 min in a well-illuminated (∼300 lux) transparent acrylic cage (40 × 40 × 15 cm). The mice were gently placed on the center and left to explore the area for 5 min. The digitized image of the path taken by each mouse was tracked by camera, and total running distances (locomotor activity) and spending times in center were analyzed using ANY-maze software. The testing apparatus was thoroughly cleaned with 70% ethanol and then dried between each animal.

### Drugs

YJ was processed and purified as described in our previous work [16]. Briefly, the medicinal plants used to prepare YJ were *Cyperus rotundus L., Ligusticum chuanxiong Hort., Gardenia jasminoides Ellis., Atractylodes lancea(Thunb.) DC.*, and *Massa fermentata*. All medicinal plants were purchased from Nanjing GuoYi Clinical, Medicinal Material Department (Nanjing, China). The herbal mixture was powdered, immersed in 95% of ethanol with constant shaking, and then filtered. This procedure was repeated three times, and the collected solvent was evaporated at low pressure and medium temperature (<55°C) until ethanol was completely eliminated. The extract of YJ was dispersed in Tween 80 solution (0.5%, w/v in saline) and administrated intragastrically (270 mg/ml, i.g.). Quality control of the preparation was performed as described previously using HPLC fingerprint analysis. Different samples of YJ preparation were revealed very similar and suitable [16].

Other drugs used in our study were L-NAME, a non-specific NOS inhibitor, 7-NI (7-nitroinidazole), an nNOS specific inhibitor, L-arginine, NO precursor, methylene blue (MB) as inhibitor of both NOS and sGC, NMDA as NMDA receptor agonist, MK-801 as NMDA receptor antagonist, Sildenafil, a phosphodiesterase 5 inhibitor, and they were purchased from Sigma, St. Louis, MO, U.S.A. Drugs solution was made in saline (0.9%). Intraperitoneal (i.p.) route was followed for all drugs administration in constant volume of 10 ml/kg of body weight.

### Drugs treatment

To investigate the involvement of NO-cGMP pathway in the antidepressant-like effect produced by YJ, MK-801 (0.05 mg/kg) [9], L-NAME (10 mg/kg) [34], MB (10 mg/kg) [35], and 7-nitroindazole (7-NI, 30 mg/kg) [21,23] were injected i.p. in mice before the administration of ineffective dosage of YJ (1.35 g/kg, half of the effective dosage, i.g.). Meanwhile, NMDA (75 mg/kg), L-arginine (750 mg/kg) [36], and sildenafil (5 mg/k) [37], were injected i.p. in mice before the administration of effective dosage of YJ (2.7 g/kg, i.g.). The whole paradigm of drug treatment and behavior test were revealed in Supplementary Figure S1 in supplemental materials. Each time point or behavior test was used independent animals.

### Statistics

All data were presented as means ± SEM. Differences amongst groups were determined using two-way ANOVA, followed by a Bonferroni’s *post hoc* analysis if appropriate. *P<*0.05 was the accepted level of significance. GraphPad Prism 6.0 (GraphPad Software, San Diego, CA, U.S.A.) was used in all statistical analyses.

## Results

### NMDA receptors involvement in the antidepressant-like effect of YJ

**Figure 1:**
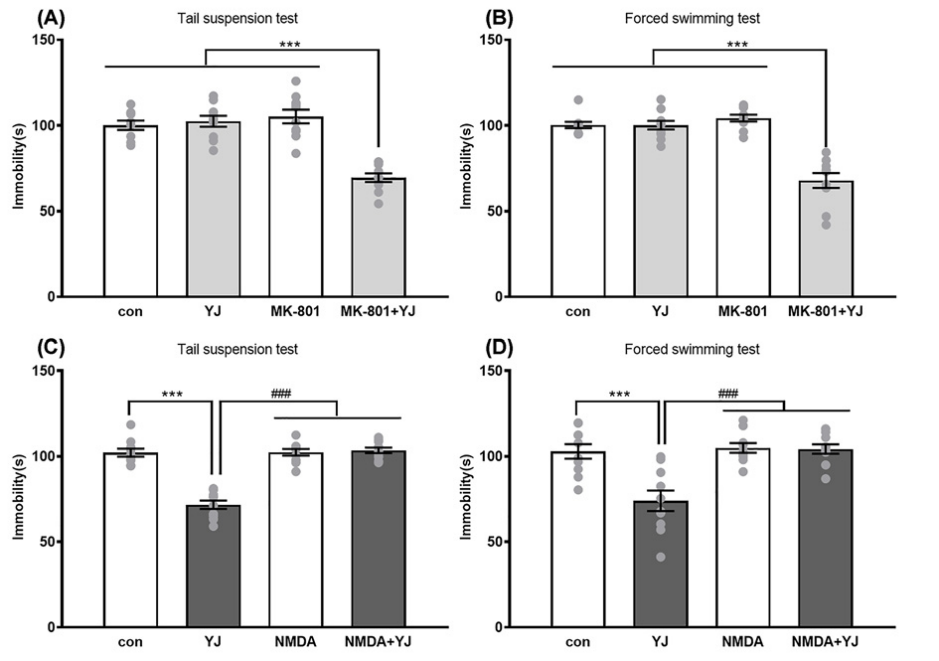
Role of NMDA receptors involved in the antidepressant-like effect of YJ in tail suspension test and forced swimming test Effect of MK-801 (0.05 mg/kg, i.p.) co-administrate with YJ (1.35 g/kg, i.g.) on immobility time in the (**A**) TST, a two-way ANOVA revealed the main effect of treatment (*F* [1, 35] = 27.41, *P*<0.001), pretreatment (*F* [1, 35] = 19.1, *P*<0.001) and of treatment × pretreatment interaction (*F* [1, 35] = 35.52, *P*<0.001) and (**B**) FST, a two-way ANOVA revealed the main effect of treatment (*F* [1, 36] = 40.86, *P*<0.001), pretreatment (*F* [1, 36] = 24.35, *P*<0.001) and of treatment × pretreatment interaction (*F* [1, 36] = 40.23, *P*<0.001). ***P<0.001 compared with control group/YJ alone group/MK-801 alone group. Effect of NMDA (75 mg/kg, i.p.) pretreatment on antidepressant effect of YJ (2.7 g/kg, i.g) in the (**C**) TST (A two-way ANOVA revealed the main effect of treatment (*F* [1, 36] = 48.63, *P*<0.001), pretreatment (*F* [1, 36] = 58.22, *P*<0.001) and of treatment × pretreatment interaction (*F* [1, 36] = 56.35, *P*<0.001) and (**D**) FST(A two-way ANOVA revealed the main effect of treatment (*F* [1, 36] = 12.43, *P*<0.01), pretreatment (*F* [1, 36] = 14.95, *P*<0.001) and of treatment × pretreatment interaction (*F* [1, 36] = 11.35, *P*<0.01). ***P<0.001 compared with control group; or ^###^*P*<0.001 with NMDA alone or with YJ group. The immobility duration in TST or FST for last 4 min of the test. Scores are expressed as the mean ± SEM, *n*=9–10 animals/group and were analyzed using two-way ANOVA followed by Bonferroni’s *post hoc* test.

In order to test whether NMDA receptors were participated in the antidepressant-like effect of YJ, MK-801, an NMDA receptors antagonist were first pretreated. About 45 min after MK-801 (0.05 mg/kg, i.p.) administration, YJ (1.35 g/kg, i.g.) was injected, and then 30 min later, the behavioral test was carried out. *Post hoc* analyses indicated that neither in TST (Figure 1A) nor in FST (Figure 1B), non-effect dosage of MK-801 or YJ affects the immobility time. However, coupled with YJ and MK-801, they could significantly decrease the immobility time in TST (*P<*0.001) compared with control group, single MK-801 group and single YJ group as well as in FST (*P<*0.001). Fig S2a and S2b showed MK-801 and YJ alone or in combination both had none effect on the locomotor activity.

Ketamine has higher affinity as an NMDA channel blocker to decrease the activity of NMDA receptors to cause antidepressant-like activity [38]. Then we also tested the effect when co-administration of NMDA and YJ on immobility time during TST (Figure 1C) and FST (Figure 1D). About 30 min after ineffective dosage of NMDA (75 mg/kg, i.p.) administration, YJ (2.7 g/kg, i.g.) was injected, and then 30 min later, the behavioral test was measured. *Post hoc* analyses indicated that NMDA alone could not affect the immobility time during behavior test. However, when pretreated with NMDA, it could block the antidepressant-like effect of YJ during TST (*P<*0.001) and FST (*P<*0.001). The NMDA or co-administration with YJ is not due to the generalized increases in locomotor activity in the present studies (Supplementary Figure S2C,D).

### NO involvement in the antidepressant-like effect of YJ

**Figure 2:**
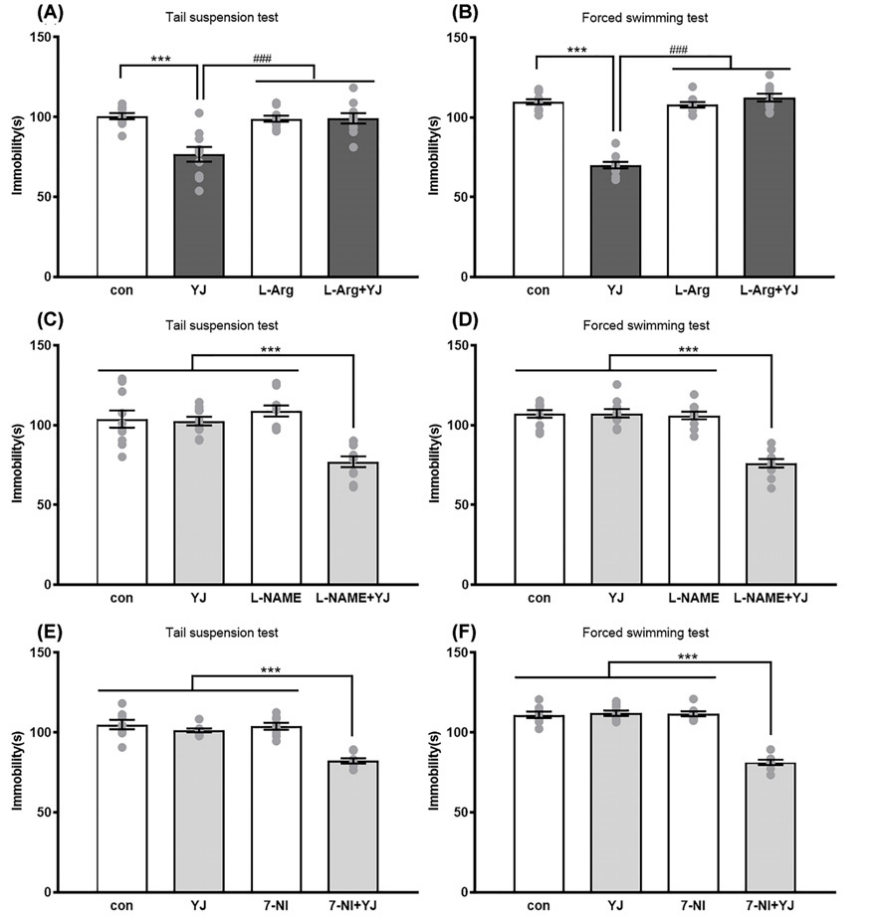
The effect of L-arg, L-NAME and 7-NI on antidepressant-like response of YJ in mice Effect of L-Arg (750 mg/kg, i.p.) pretreatment on antidepressant effect of YJ (2.7 g/kg, i.g) in the (**A**) TST, a two-way ANOVA revealed the main effect of treatment (*F* [1, 36] = 14.53, *P*<0.01), pretreatment (*F* [1, 36] = 11.33, *P*<0.001) and of treatment × pretreatment interaction (*F* [1, 36] = 15.05, *P*<0.001) and (**B**) FST, a two-way ANOVA revealed the main effect of treatment (*F* [1, 36] = 78.25, *P*<0.001), pretreatment (*F* [1, 36] = 104, *P*<0.001) and of treatment × pretreatment interaction (*F* [1, 36] = 123.8, *P*<0.001). ***P<0.001 compared with control group; or ^###^*P*<0.001 with L-Arg alone or with YJ group. Effect of L-NAME (10 mg/kg, i.p.) co-administrate with YJ (1.35 g/kg, i.g.) on immobility time in the (**C**) TST, a two-way ANOVA revealed the main effect of treatment (*F* [1, 36] = 18.49, *P*<0.001), pretreatment (*F* [1, 36] = 7.018, *P*<0.05) and of treatment × pretreatment interaction (*F* [1, 36] = 15.73, *P*<0.001) and (**D**) FST, a two-way ANOVA revealed the main effect of treatment (*F* [1, 36] = 34.62, *P*<0.001), pretreatment (*F* [1, 36] = 41.31, *P*<0.05) and of treatment × pretreatment interaction (*F* [1, 36] = 36.23, *P*<0.001). Effect of 7-NI (30 mg/kg, i.p.) co-administrate with YJ (1.35 g/kg, i.g.) on immobility time in the (**E**) TST, a two-way ANOVA revealed the main effect of treatment (*F* [1, 28] = 36.67, *P*<0.001), pretreatment (*F* [1, 28] = 23.24, *P*<0.001) and of treatment × pretreatment interaction (*F* [1, 28] = 18.74, *P*<0.001) and (**F**) FST, a two-way ANOVA revealed the main effect of treatment (*F* [1, 28] = 72.26, *P*<0.001), pretreatment (*F* [1, 28] = 76.37, *P*<0.001) and of treatment × pretreatment interaction (*F* [1, 28] = 82.17, *P*<0.001). The immobility duration in TST and FST for last 4 min of the test. Scores are expressed as the mean ± SEM, *n*=10 animals/group and were analyzed using two-way ANOVA followed by Bonferroni’s *post hoc* test. ***P<0.001 compared with saline treated vehicle; or ^###^*P*<0.001 with YJ treated group.

*Post hoc* analyses indicated that pretreatment of mice with L-arginine (750 mg/kg, i.p.) for 60 min could halt the antidepressant-like effect of YJ (2.7 g/kg, i.g.) treatment for 30 min during TST (*P<*0.001, Figure 2A). Meanwhile, during FST paradigm, the same results indicated that L-arginine also blocked the antidepressant-like effects of YJ (*P<*0.001, Figure 2B). When combined L-arginine with YJ, they could not affect the locomotor activity in OFT (Supplementary Figure S3A,B).

Figure 2C shows the effects of co-administration of L-NAME (10 mg/kg, i.p.) and YJ (1.35 g/kg, i.g.) on the immobility time during behavior test. L-NAME alone did not cause any effect on the immobility time either in TST or in FST. However, when pretreated with the same dose of L-NAME, it could combine YJ to induce the decrease of immobility time both in TST (*P<*0.001, Figure 2C) and FST (*P<*0.001, Figure 2D). L-arginine when co-administrated with either saline or YJ, failed to change the locomotor activity of mice during OFT (Supplementary Figure S3C,D).

Furthermore, we tested whether the NOS activity involved in the antidepressant-like effect of YJ. 7-NI (30 mg/kg i.p.), a specific neuronal NOS inhibitor, when treated for 30 min before ineffective YJ (1.35 g/kg, i.g.) also displayed antidepressant-like activity in TST (*P<*0.001, Figure 2E). *Post hoc* analyses also indicated that the similar trend in FST (*P<*0.001, Figure 2F). Pretreatment with sildenafil alone or combined with YJ, both failed to elicit any effect on immobility time (Supplementary Figure S3E,F).

### Involvement of cGMP in the antidepressant-like activity of YJ during depressive test

**Figure 3:**
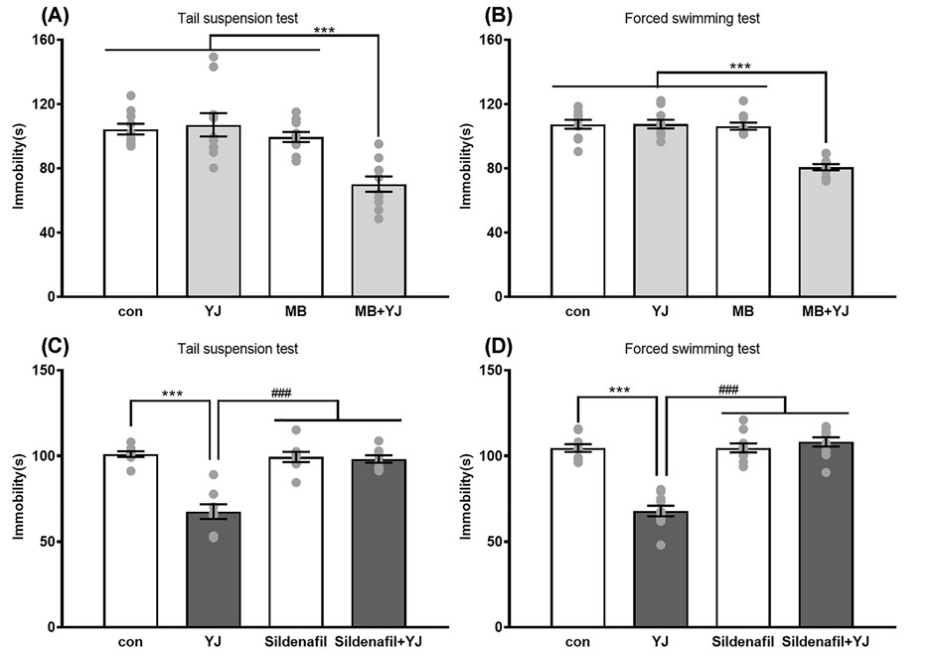
The effect of MB and Sildenafil on antidepressant-like effect of YJ in tail suspension test and forced swimming test in mice Effect of MB (10 mg/kg, i.p.) co-administrate with YJ (1.35 g/kg, i.g.) on immobility time in the (**A**) TST, a two-way ANOVA revealed the main effect of treatment (*F* [1, 36] = 7.432, *P<*0.01), pretreatment (*F* [1, 36] = 18.24, *P*<0.001) and of treatment × pretreatment interaction (*F* [1, 36] = 10.71, *P*<0.01) and (**B**) FST, a two-way ANOVA revealed the main effect of treatment (*F* [1, 36] = 27.31, *P*<0.001), pretreatment (*F* [1, 36] = 32.94, *P*<0.001) and of treatment × pretreatment interaction (*F* [1, 36] = 27.92, *P*<0.001). Effect of Sildenafil (5 mg/kg, i.p.) pretreatment on antidepressant effect of YJ (2.7 g/kg, i.g.) in the (**C**) TST, a two-way ANOVA revealed the main effect of treatment (*F* [1, 28] = 34.63, *P*<0.001), pretreatment (*F* [1, 28] = 24.16, *P*<0.05) and of treatment × pretreatment interaction (*F* [1, 28] = 29.92, *P*<0.001) and (**D**) FST, a two-way ANOVA revealed the main effect of treatment (*F* [1, 36] = 39.12, *P*<0.001), pretreatment (*F* [1, 36] = 58.02, *P*<0.05) and of treatment × pretreatment interaction (*F* [1, 36] = 57.22, *P*<0.001). The immobility duration in TST and FST for last 4 min of the test. Scores are expressed as the mean ± SEM, *n*=10 animals/group and were analyzed using two-way ANOVA followed by Bonferroni’s *post hoc* test. ***P<0.001 compared with saline treated vehicle; or ^###^*P*<0.001 with YJ treated group.

MB could inhibit both NOS and sGC, while MB (10 mg/kg, i.p.) was administrated alone was unable to affect immobility time during TST or FST. However, MB (administration before 60 min) was combined with ineffective dose of YJ (1.35 g/kg, i.g.) was enough to decrease immobility time during TST (Figure 3A) and FST (Figure 3B).

In order to investigate the function of cGMP in the antidepressant-like effects of YJ, mice were pre-treated with sildenafil (5 mg/kg, i.p.), administered 30 min before the second administration of YJ (2.7g/kg, i.g.). Sildenafil was able to reverse the antidepressant effect of YJ in TST (Figure 3C) and FST (Figure 3D).

## Discussion

In current study, we examined the role of NMDA receptors and L-arginine/NO/cGMP pathway in the antidepressant-like activity of YJ. NMDA could block the antidepressant effect of YJ while MK801, an NMDA receptors antagonist, boosted the effect of ineffective dosage of YJ. Though we did not investigate the accurate mechanism from our study through which YJ interacted with NMDA receptor. Yet, it recommends that such effect of YJ is reliant on the blockage of NMDA receptor. Nevertheless, there was strong interplay between NMDA and NO in the antidepressant-like effects of certain drugs [39]. Undeniably, the effect of cGMP in depression was also well understood, as certain studies had shown that NO and cGMP were involved in the initiation of depression. Drugs which inhibited the L-arginine and NO-cGMP signaling were reported to have antidepressant-like effects [40]. Similarly, co-administration with L-Arg, an NO precursor, and sildenafil, a PDE5 inhibitor, both could blunt the effect of YJ, whereas inhibition of L-arginine/NO/cGMP signaling pathway by 7-NI, L-NAME, or MB, all facilitated antidepressant-like effect of YJ. The study first demonstrated that YJ’s antidepressant-like effect is mostly dependent on NMDA/NO/cGMP signaling pathway.

In the present study, we first evaluated the dependence of NMDA receptors on the antidepressant-like effects of YJ in TST and FST behavior. Previous work indicated that prior administration of NMDA could reverse the antidepressant-like effect of SSRIs [7,9,29,30]. NMDA receptor is also profoundly implicated in pathology of depression and involved in antidepressant activity such as ketamine [41]. Indeed, results from our previous data supported that the selective decrease in NR1 by YJ, in contrast with the selectively increased NR1 in the PFC of chronically stressed mice [32]. Meanwhile, we also found that YJ induced an instant and lasting down-regulation of NR1 expression in the hippocampus [18]. It indicated that in physiological condition and pathological condition NMDA receptors display different function. Taken together, the attainment of antidepressant-like effect of YJ by MK-801 implied that antidepressant-like effect is at least in part achieved via the down-regulation of NMDA receptors. Meanwhile, we further confirmed that anti-immobility property in mice is reversed by pretreatment with NMDA. These were also consistent with the antidepressant-like phenotype in knockout mice with subunits of NR2A or NR2B in NMDA receptor [42,43]. Therefore, NMDA receptors activity is likely, at least in part, responsible for antidepressant effect of YJ.

Following the activation of NMDA receptors, NO is produced from L-arginine. Meanwhile, NMDA receptors enhance sGC activity, which produces cGMP, a mediator of the effects of NO [25]. The interaction between NMDA and NO in antidepressant-like effects of medicine, led to study the effect of NOS in depression [44]. Some drugs were reported to display antidepressant-like effects via inhibit the L-Arg and NO-cGMP signaling during FST [29,45–47]. In the etiology of depression, many studies supported that L-Arg/NO/cGMP pathway is participated in the depressive behavior [21,48]. In current study, we found that pretreatment with L-Arg can reverse the antidepressant-like effect of YJ both in TST and FST, but not affect the locomotor activity of mice. In our another study, NO concentration was rapidly decreased after YJ treatment [49], and it indicated that YJ regulate the activity of NMDA receptors to affect NO synthesis. Similarly, another study reported that NMDA receptor modulates the activity of NOS and accelerates the formation of NO [50,51]. Above all, it is likely that YJ developes antidepressant-like effect by inhibition of NO synthesis. In order to confirm our hypothesis that YJ dependent on the NOS activity to show antidepressant-like effect, mice were pretreated with ineffective dose of 7-NI or L-NAME. Both 7-NI and L-NMAE combined with YJ produced antidepressant-like effect. Clinical widely used medicines such as fluoxetine, imipramine [44], and venlafaxine [35] were also reported to enhance the antidepressant-like effect with 7-NI. Our results demonstrated that ineffective YJ could decrease the immobility time in mice with 7-nitroindazole and MB treatment, which was consistent with other researches [52–54].

The excitatory effect of NMDA is due in part to formation of NO and cGMP. Our recent data are reinforced that YJ produces a noticeable reduction in cGMP levels and it may be the important target which manifests its antidepressant-like effect [49]. It is considered that NO exerts its neurotransmitter effect via sGC, while sGC can transfer GTP to cGMP [25]. Then we investigate that MB (both NOS and sGC inhibitor) co-administration with YJ displays significant antidepressant-like effect, while single administration has no function on immobility time in depressive test. Venlafaxine also enhanced antidepressant-like effect combined with MB [35]. The sGC regulated the cGMP concentration, while cGMP was also dependent on PDE5, which was involved in catalyzing cGMP to GMP [55,56]. Sildenafil (a PDE5 inhibitor) could increase the level of cGMP [55], and it reversed antidepressant-like effect of YJ in the present study. It indicated that YJ depends on the NOS and sGC activity. Venlafaxine [35] and Lithium [57] also demonstrated that cGMP could play key role in antidepressant-like effect of them.

Hence, summing up the results, it can be concluded that NMDA receptors and L-arginine-NO cGMP pathway is involved in the antidepressant-like mechanism of YJ.

## Supporting information

**Supplementary Figure S1:** The paradigm of the whole experiment about the drug treatments and behavior tests.

**Supplementary Figure S2:** Effect of MK-801 (0.05mg/kg, i.p.) co-administrate with YJ (1.35g/kg, i.g.) on locomotor activity (**a**) and anxiety (**b**) in the open field test. Effect of NMDA (75mg/kg, i.p.) pretreatment on locomotor activity (**c**) and anxiety (**d**) of YJ (2.7g/kg, i.g) in the open field test. The total distance and central time recorded during open-field test. Scores are expressed as the mean ± SEM, n=10 animals/group and were analyzed using two-way ANOVA followed by Bonferroni’s post-test.

**Supplementary Figure S3:** Effect of L-Arg (750mg/kg, i.p.) pretreatment on locomotor activity (**a**) and anxiety (**b**) of YJ (2.7g/kg, i.g.) in the open field test. Effect of L-NAME (10mg/kg, i.p.) co-treatment of YJ (1.35g/kg, i.g) on locomotor activity (**c**) and anxiety (**d**) in the open field test. Effect of 7-NI (30mg/kg, i.p.) co-treatment of YJ (1.35g/kg, i.g) on locomotor activity (**e**) and anxiety (**f**) in the open field test. The total distance and central time recorded during open-field test. Scores are expressed as the mean ± SEM, n=10 animals/group and were analyzed using two-way ANOVA followed by Bonferroni’s post-test.

**Supplementary Figure S4:** Effect of MB (10mg/kg, i.p.) co-administrate with YJ (1.35g/kg, i.g.) on locomotor activity (**a**) and anxiety (**b**) in the open field test. Effect of sildenafil (5mg/kg, i.p.) pretreatment on locomotor activity (**c**) and anxiety (**d**) of YJ (2.7g/kg, i.g) in the open field test. The total distance and central time recorded during open-field test. Scores are expressed as the mean ± SEM, n=10 animals/group and were analyzed using two-way ANOVA followed by Bonferroni’s post-test.

## Abbreviations

cGMP: cyclic guanosine monophosphate
FST: forced swimming test
IP: intraperitoneal
MB: methylene blue
MDD: major depressive disorder
NO: nitric oxide
SSRI: selective serotonin reuptake inhibitor
TST: tail suspension test
YJ: Yueju pill
