# Supplementary material for: NMDA receptors and L-arginine/nitric oxide/cyclic guanosine monophosphate pathway contribute to the antidepressant-like effect of Yueju pill in mice
Source: Biosci Rep. 2019 Sep 16;39(9):BSR20190524. doi: 10.1042/BSR20190524 (PMC6746996; doi:10.1042/BSR20190524)
Supplement: Supplementary file 1 [file bsr20190524_Supp1.pdf]

Supplemental materials

To assess the effect of NMDA receptors in the antidepressant-like effect of Yueju pill (YJ), we administered MK-801 (0.05mg/kg, i.p.)(9) 45 mins prior to the injection of YJ (1.35g/kg, i.g.), tail suspension test (TST), forced swimming test (FST) and open filed test (OFT) were performed after YJ treatment for 30 mins. Meanwhile, we also treat NMDA (75 mg/kg, i.p.) 30 mins prior to the injection of YJ (2.7g/kg, i.g.), TST, FST and OFT were performed after YJ treatment for 30 mins. Each behavior test used independent 32-40 mice.

In another experiment, to investigate the role of NO in YJ -mediated antidepressant-like effect, we first administered L-Arginine (750 mg/kg, i.p.) 60 mins prior to the injection of YJ (2.7g/kg, i.g.), behavior tests were performed after YJ treatment for 30 mins. Then we injected L-NAME (10 mg/kg, i.p.) 45 mins prior to the treatment of YJ (1.35g/kg, i.g.), behavior tests were performed after YJ treatment for 30 mins. Furthermore, we treated (7-NI, 30 mg/kg, i.p.) 30 mins prior to the injection of YJ (1.35g/kg, i.g.), behavior tests were performed after YJ treatment for 30 mins.

Finally, to evaluate the function of cGMP in antidepressant-like effect produced by YJ, methylene blue (10mg/kg, i.p.) was injected in mice 60 mins before the administration of YJ (1.35g/kg, i.g.), behavior tests were performed after YJ treatment for 30 mins. Also, sildenafil (5mg/kg, i.p.) was injected in mice 30 mins before the administration of YJ (1.35g/kg, i.g.), behavior tests were performed after YJ treatment for 30 mins. All the procedure was described in **Figure S1**.

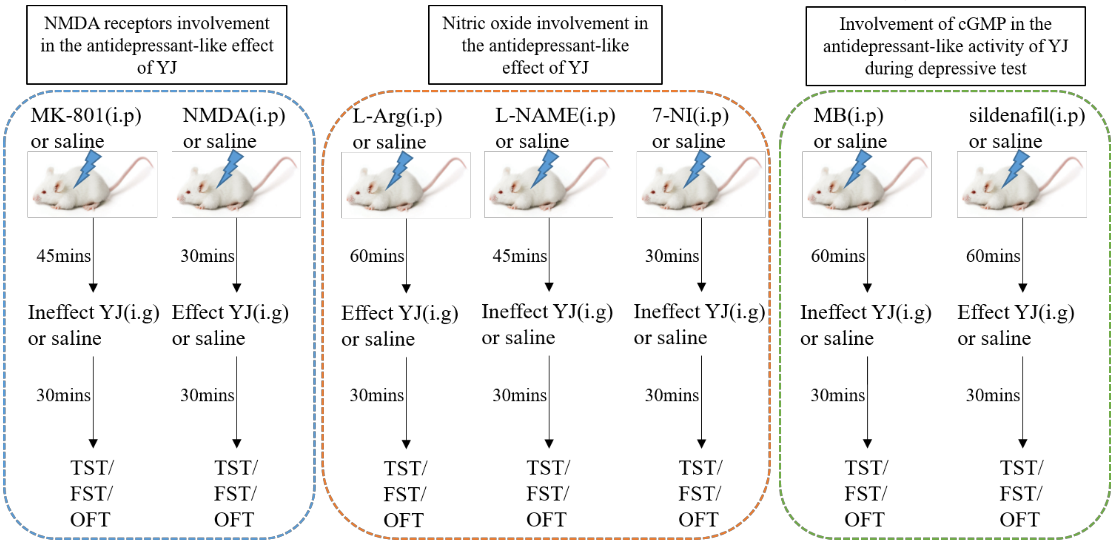

Fig. S1. The paradigm of the whole experiment about the drug treatments and behavior tests.

Injection of MK-801 alone or when combined with YJ did not affect the locomotor activity (**Fig. S2a**, A two-way ANOVA revealed the main effect of treatment (F (1, 36) = 0.348, P = 0.5589), pre-treatment (F (1, 36) = 0.02288, P = 0.8806) and of treatment × pre-treatment interaction (F (1, 36) = 0.02022, P = 0.8877)) or central time (**Fig. S2b**, A two-way ANOVA revealed the main effect of treatment (F (1, 36) = 1.172, P = 0.2863), pre-treatment (F (1, 36) = 0.004268, P = 0.9483) and of treatment × pre-treatment interaction (F (1, 36) = 0.0005566, P = 0.9813)) during OFT.

NMDA alone or when combined with effective doses of YJ (2.7g/kg), failed to elicit any effect on ambulatory activity of mice during OFT (**Fig. S2c**, A two-way ANOVA revealed the main effect of treatment (F (1, 36) = 0.001282, P = 0.9580), pre-treatment (F (1, 36) = 0.02302, P = 0.8803) and of treatment × pre-treatment interaction (F (1, 36) = 0.1316, P = 0.7189); **Fig. S2d**, A two-way

ANOVA revealed the main effect of treatment (*F* (1, 36) = 0.4899, *P* = 0.4885), pre-treatment (*F* (1, 36) = 0.8141, *P* = 0.3729) and of treatment × pre-treatment interaction (*F* (1, 36) = 0.1491, *P* = 0.7017)).

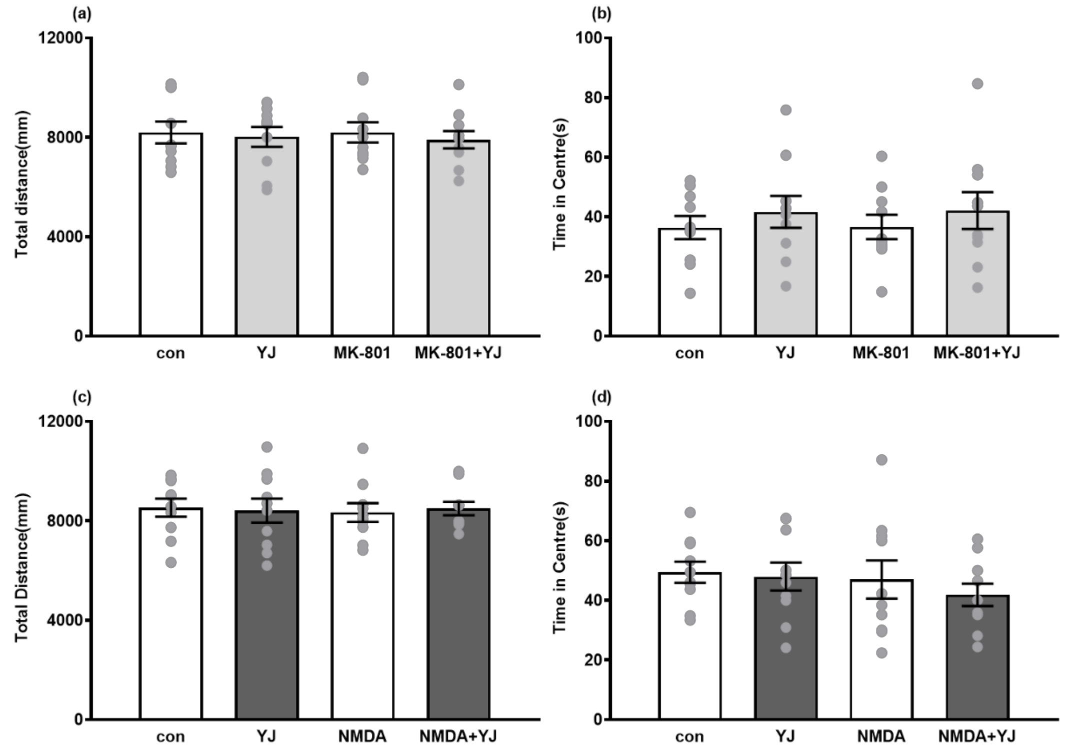

Fig. S2. Effect of MK-801 (0.05mg/kg, i.p.) co-administrate with YJ (1.35g/kg, i.g.) on locomotor activity (**a**) and anxiety (**b**) in the open field test. Effect of NMDA (75mg/kg, i.p.) pretreatment on locomotor activity (**c**) and anxiety (**d**) of YJ (2.7g/kg, i.g) in the open field test. The total distance and central time recorded during open-field test. Scores are expressed as the mean ± SEM, n=10 animals/group and were analyzed using two-way ANOVA followed by Bonferroni's post-test.

Injection of L-Arginine alone or when combined with YJ failed to affect the locomotor activity (**Fig. S3a**) during OFT. A two-way ANOVA revealed the main effect of treatment (*F* (1, 36) = 0.6924, *P* = 0.4108), pre-treatment (*F* (1, 36) = 0.5367, *P* = 0.4685) and of treatment × pre-treatment interaction (*F* (1, 36) = 2.03, *P* = 0.1628). Meanwhile, L-Arginine alone or combined with YJ also didn't affect the anxiety behavior (central time) during OFT (**Fig. S3b**). A two-way ANOVA revealed the main effect of treatment (*F* (1, 36) = 0.2202, *P* = 0.6417), pre-treatment (*F* (1, 36) = 1.849, *P* = 0.1823) and of treatment × pre-treatment interaction (*F* (1, 36) = 1.181, *P* = 0.2845).

Fig. S3c and **S3d** clearly validated that co-injection of YJ (1.35g/kg) with L-NAME failed to bring any significant changes in locomotor behavior [**Fig. S3c**, A two-way ANOVA revealed the main effect of treatment (*F* (1, 36) = 1.449, *P* = 0.2365), pre-treatment (*F* (1, 36) = 1.815, *P* = 0.1864) and of treatment × pre-treatment interaction (*F* (1, 36) = 0.007419, *P* = 0.9318)] or anxiety behavior [**Fig. S3d**, A two-way ANOVA revealed the main effect of treatment (*F* (1, 36) = 1.978, *P* = 0.1681), pre-treatment (*F* (1, 36) = 1.581, *P* = 0.2168) and of treatment × pre-treatment interaction (*F* (1, 36) = 0.1624, *P* = 0.6893)] of animals during OFT.

Fig. S3e and **Fig. S3f** revealed the effects of 7-NI administration alone or with YJ combination. No significant change was found in locomotor activity [A two-way ANOVA revealed the main effect of

treatment (F (1, 28) = 0.0489, P = 0.8266), pre-treatment (F (1, 28) = 0.3187, P = 0.5769) and of treatment × pre-treatment interaction (F (1, 28) = 0.249, P = 0.6217)] or anxiety behavior [A two-way ANOVA revealed the main effect of treatment (F (1, 28) = 0.081, P = 0.7780), pre-treatment (F (1, 28) = 0.1596, P = 0.6925) and of treatment × pre-treatment interaction (F (1, 28) = 0.04017, P = 0.8426)] during OFT.

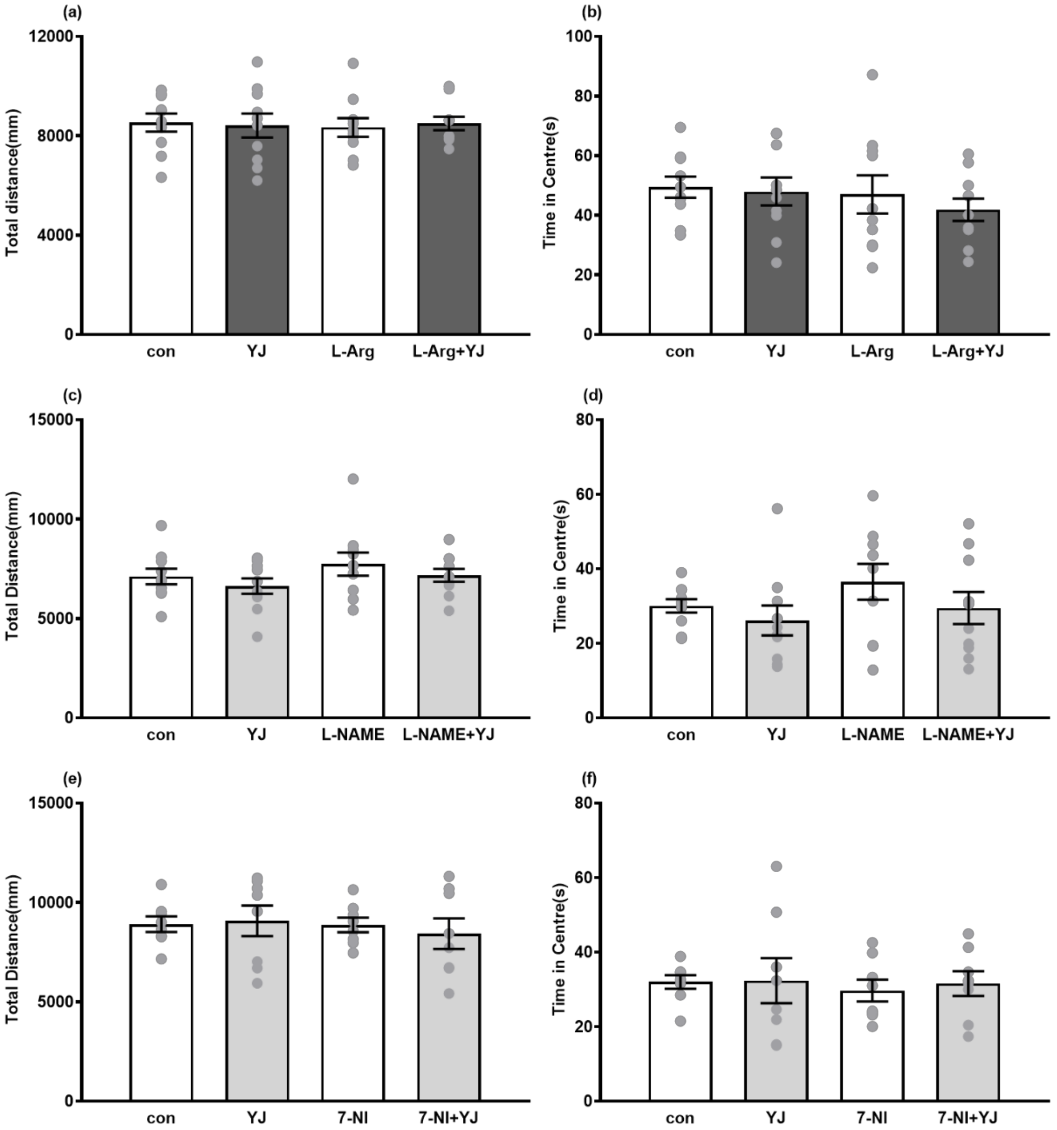

Fig. S3. Effect of L-Arg (750mg/kg, i.p.) pretreatment on locomotor activity (**a**) and anxiety (**b**) of YJ (2.7g/kg, i.g.) in the open field test. Effect of L-NAME (10mg/kg, i.p.) co-treatment of YJ (1.35g/kg, i.g) on locomotor activity (**c**) and anxiety (**d**) in the open field test. Effect of 7-NI (30mg/kg, i.p.) co-treatment of YJ (1.35g/kg, i.g) on locomotor activity (**e**) and anxiety (**f**) in the open field test. The total distance and central time recorded during open-field test. Scores are expressed as the mean ± SEM, n=10 animals/group and were analyzed using two-way ANOVA followed by Bonferroni's post-test.

Administration of methylene blue (MB) alone or in combination with YJ did not show any significant effect (p > 0.05) on locomotor activity [**Fig. S4a**, A two-way ANOVA revealed the main effect of treatment (F (1, 36) = 0.4803, P = 0.4927), pre-treatment (F (1, 36) = 0.105, P = 0.7478) and of treatment × pre-treatment interaction (F (1, 36) = 0.004815, P = 0.8275)] or anxiety behavior

[**Fig. S4b**, A two-way ANOVA revealed the main effect of treatment (F (1, 36) = 0.03025, P = 0.8629), pre-treatment (F (1, 36) = 0.01549, P = 0.9016) and of treatment × pre-treatment interaction (F (1, 36) = 0.007308, P = 0.9323)] during OFT.

Sildenafil administration, alone or in combination with YJ did not show any significant effect on locomotor activity [**Fig S4c**, A two-way ANOVA revealed the main effect of treatment (F (1, 36) = 1.805, P = 0.1875), pre-treatment (F (1, 36) = 1.551, P = 0.2211) and of treatment × pre-treatment interaction (F (1, 36) = 0.1085, P = 0.7437)] or central time [**Fig S4d**, A two-way ANOVA revealed the main effect of treatment (F (1, 36) = 3.265e-005, P = 0.9955), pre-treatment (F (1, 36) = 1.993, P = 0.1666) and of treatment × pre-treatment interaction (F (1, 36) = 1.46, P = 0.2349)] during OFT.

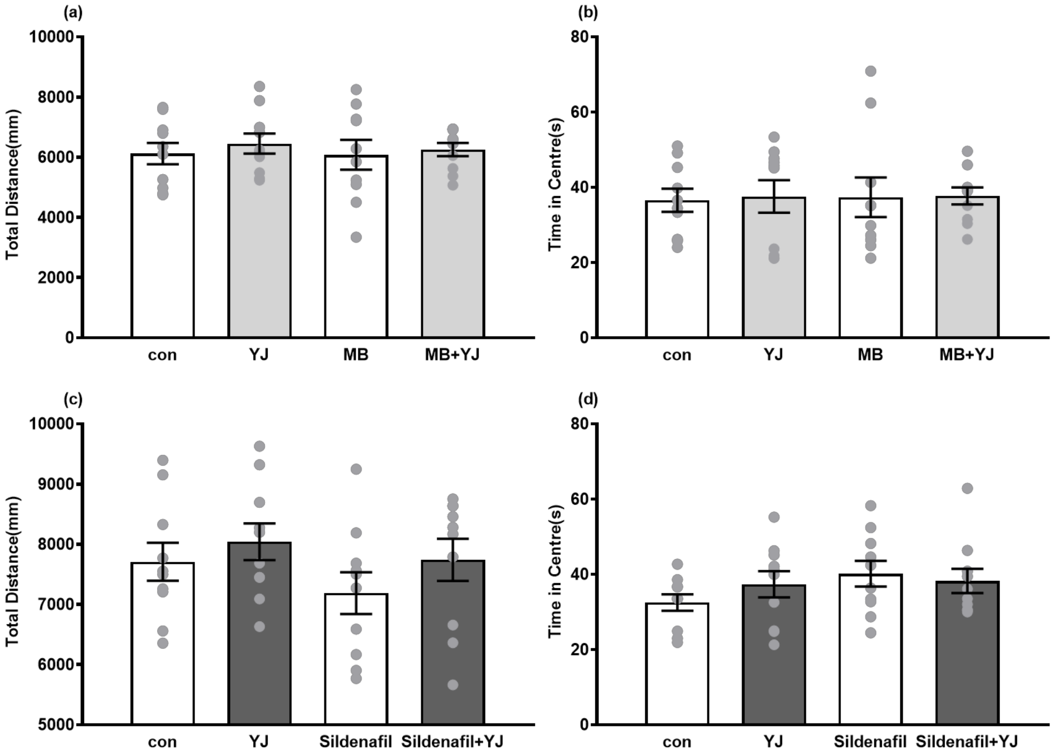

Fig. S4. Effect of MB (10mg/kg, i.p.) co-administrate with YJ (1.35g/kg, i.g.) on locomotor activity (**a**) and anxiety (**b**) in the open field test. Effect of sildenafil (5mg/kg, i.p.) pretreatment on locomotor activity (**c**) and anxiety (**d**) of YJ (2.7g/kg, i.g) in the open field test. The total distance and central time recorded during open-field test. Scores are expressed as the mean ± SEM, n=10 animals/group and were analyzed using two-way ANOVA followed by Bonferroni's post-test.
